# Bis(4-amino­benzene­sulfonato-κ*N*)diaqua­bis(dimethyl­formamide-κ*O*)nickel(II) dihydrate

**DOI:** 10.1107/S1600536809020406

**Published:** 2009-06-06

**Authors:** Jun Tan, Ya-Ping He, Xiao-Li Bao

**Affiliations:** a College of Biological and Chemical Engineering, Jiaxing University, Jiaxing 314001, People’s Republic of China

## Abstract

In the title compound, [Ni(C_6_H_6_NO_3_S)_2_(C_3_H_7_NO)_2_(H_2_O)_2_]·2H_2_O, the Ni^II^ ion (site symmetry 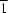) is coordinated by two –NH_2_ groups from two 4-amino­benzene­sulfonate anions, two O atoms from two dimethyl­formamide mol­ecules and two water mol­ecules, forming a slightly distorted *trans*-NiN_2_O_4_ octa­hedral geometry. In the crystal structure, inter­molecular O—H⋯O, O—H⋯(O,O) and N—H⋯O hydrogen bonds link the components into a three-dimensional network. The O atoms of the sulfonate group are disordered over two sets of sites in a 0.833 (4):0.167 (4) ratio and the O atom of the uncoordinated water mol­ecule is disordered over two sites in a 0.637 (18):0.363 (18) ratio.

## Related literature

For related structures, see: Zhao *et al.* (2007 ▶); Li *et al.* (2008 ▶).
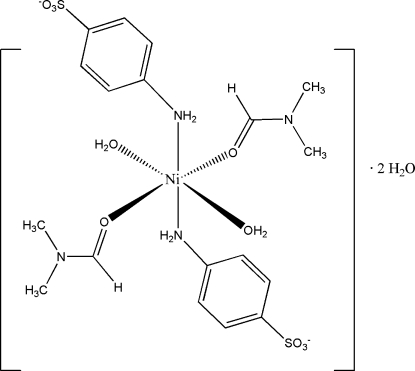


## Experimental

### 

#### Crystal data

[Ni(C_6_H_6_NO_3_S)_2_(C_3_H_7_NO)_2_(H_2_O)_2_]·2H_2_O
*M* *_r_* = 621.32
Orthorhombic, 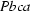

*a* = 11.3197 (6) Å
*b* = 15.2174 (7) Å
*c* = 15.9061 (8) Å
*V* = 2739.9 (2) Å^3^
*Z* = 4
Mo *K*α radiation
μ = 0.92 mm^−1^
*T* = 296 K
0.20 × 0.18 × 0.15 mm

#### Data collection

Bruker SMART CCD diffractometer
Absorption correction: multi-scan (*SADABS*; Siemens, 1996 ▶) *T*_min_ = 0.837, *T*_max_ = 0.874
13538 measured reflections
2424 independent reflections
1991 reflections with *I* > 2σ(*I*)
*R*_int_ = 0.030

#### Refinement

*R*[*F* ^2^ > 2σ(*F* ^2^)] = 0.029
*wR*(*F* ^2^) = 0.075
*S* = 1.03
2424 reflections
209 parameters
H-atom parameters constrained
Δρ_max_ = 0.26 e Å^−3^
Δρ_min_ = −0.22 e Å^−3^

### 

Data collection: *SMART* (Siemens, 1996 ▶); cell refinement: *SAINT* (Siemens, 1996 ▶); data reduction: *SAINT*; program(s) used to solve structure: *SHELXS97* (Sheldrick, 2008 ▶); program(s) used to refine structure: *SHELXL97* (Sheldrick, 2008 ▶); molecular graphics: *SHELXTL* (Sheldrick, 2008 ▶); software used to prepare material for publication: *SHELXTL*.

## Supplementary Material

Crystal structure: contains datablocks global, I. DOI: 10.1107/S1600536809020406/hb2985sup1.cif

Structure factors: contains datablocks I. DOI: 10.1107/S1600536809020406/hb2985Isup2.hkl

Additional supplementary materials:  crystallographic information; 3D view; checkCIF report

## Figures and Tables

**Table 1:** Selected bond lengths (Å)

Ni1—O4	2.0385 (15)
Ni1—O5	2.0664 (15)
Ni1—N1	2.1579 (19)

**Table 2:** Hydrogen-bond geometry (Å, °)

*D*—H⋯*A*	*D*—H	H⋯*A*	*D*⋯*A*	*D*—H⋯*A*
N1—H1*A*⋯O1^i^	0.90	2.15	2.992 (3)	156
N1—H1*B*⋯O3^ii^	0.90	2.06	2.919 (3)	160
O5—H5*B*⋯O6*A*^i^	0.85	1.84	2.685 (5)	174
O5—H5*B*⋯O6*B*^i^	0.85	1.87	2.669 (8)	156
O5—H5*C*⋯O3^iii^	0.85	1.95	2.743 (3)	155
O6*A*—H6*A*⋯O2^iv^	0.85	1.96	2.768 (6)	158
O6*B*—H6*B*⋯O1	0.85	1.95	2.694 (8)	146

Symmetry codes: (i) 
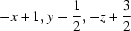
; (ii) 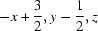; (iii) 
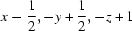
; (iv) 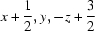.

**Fig. 1.**
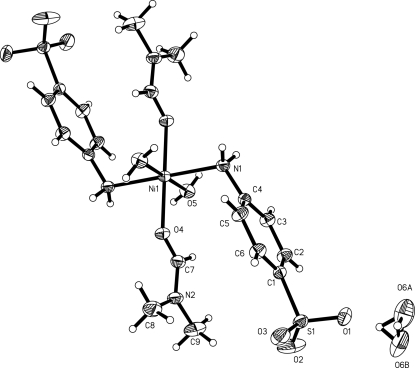
The molecular structure of (I), with displacement ellipsoids drawn at the 30% probability level. Unlabelled atoms in the complex are generated by the symmetry operation (1–x, –y, 1–z).

**Fig. 2.**
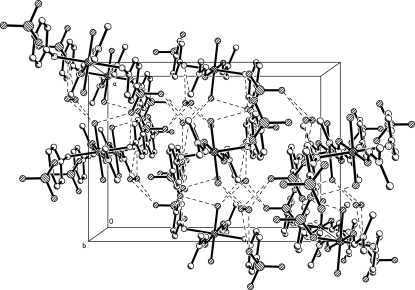
Part of the crystal structure of the title compound showing hydrogen bonds as dashed lines.

## supplementary crystallographic information

### Comment

4-Aminobenzenesulfonic acid can bind to transition metals through the amino as well as the carboxylate groups (Zhao *et al.*, 2007; Li *et al.*, 2008). Therefore, we extended these investigations to the use of the ligand 4-aminobenzenesulfonic acid and obtained various framework structures.

In this paper, we report the structure of the title compound, (I), im which the Ni^II^ ion is located on a crystallographic inversion center and is coordinated by two –NH_2_ groups from two 4-aminobenzenesulfonate ligands and four oxygen atoms from two water molecules and two *N*,*N*'-dimethylformamide molecules (Table 1 and Fig. 1), forming a slightly distorted octahedral coordination environment.

In the crystal structure, intermolecular O—H···O and N—H···O hydrogen bonds link the title complex into a three-dimensional network (Table 2 and Fig.2).

### Experimental

An ethanol solution (20 ml) containing nickel chloride (0.237 g, 1 mmol) was added dropwise to an aqueous solution containing 4-aminobenzenesulfonic acid (0.180 g, 1 mmol) and sodium hydroxide (0.040 g, 1 mmol) with stirring over a period of 10 min. The green solid compound was separated out and dissolved in *N*,*N*-dimethylformamide, then the green solution was filtrated. After 20 days, green blocks of (I) were produced from the filtrate (yield: 35.3%).

### Refinement

The –SO_3_ group is disordered over two positions with respect to the O atoms in a 0.83 (1):0.17 (1) ratio. The solvent water molecule is also disordered over two positions in a 0.64 (4):0.46 (4) ratio. All H atoms were initially located in a difference map, then relocated to idealised positions (C—H = 0.93–0.96 Å, O—H = 0.85 Å, N—H = 0.90 Å) and refined as riding with *U*_iso_(H) = 1.2*U*_eq_(C,*N*) or 1.5*U*_eq_(O).

### Crystal data

**Table:**

[Ni(C_6_H_6_NO_3_S)_2_(C_3_H_7_NO)_2_(H_2_O)_2_]·2H_2_O	*F*(000) = 1304
*M**_r_* = 621.32	*D*_x_ = 1.506 Mg m^−^^3^
Orthorhombic, *P**b**c**a*	Mo *K*α radiation, λ = 0.71073 Å
Hall symbol: -P 2ac 2ab	Cell parameters from 4096 reflections
*a* = 11.3197 (6) Å	θ = 2.6–25.5°
*b* = 15.2174 (7) Å	µ = 0.92 mm^−^^1^
*c* = 15.9061 (8) Å	*T* = 296 K
*V* = 2739.9 (2) Å^3^	Block, green
*Z* = 4	0.20 × 0.18 × 0.15 mm

### Data collection

**Table:**

Bruker SMART CCD diffractometer	2424 independent reflections
Radiation source: fine-focus sealed tube	1991 reflections with *I* > 2σ(*I*)
graphite	*R*_int_ = 0.030
ω scans	θ_max_ = 25.0°, θ_min_ = 2.6°
Absorption correction: multi-scan (*SADABS*; Siemens, 1996)	*h* = −12→13
*T*_min_ = 0.837, *T*_max_ = 0.874	*k* = −16→18
13538 measured reflections	*l* = −18→18

### Refinement

**Table:**

Refinement on *F*^2^	Primary atom site location: structure-invariant direct methods
Least-squares matrix: full	Secondary atom site location: difference Fourier map
*R*[*F*^2^ > 2σ(*F*^2^)] = 0.029	Hydrogen site location: inferred from neighbouring sites
*wR*(*F*^2^) = 0.075	H-atom parameters constrained
*S* = 1.03	*w* = 1/[σ^2^(*F*_o_^2^) + (0.0336*P*)^2^ + 1.5689*P*] where *P* = (*F*_o_^2^ + 2*F*_c_^2^)/3
2424 reflections	(Δ/σ)_max_ = 0.001
209 parameters	Δρ_max_ = 0.26 e Å^−^^3^
0 restraints	Δρ_min_ = −0.22 e Å^−^^3^

### Special details

**Table:**

Geometry. All e.s.d.'s (except the e.s.d. in the dihedral angle between two l.s. planes) are estimated using the full covariance matrix. The cell e.s.d.'s are taken into account individually in the estimation of e.s.d.'s in distances, angles and torsion angles; correlations between e.s.d.'s in cell parameters are only used when they are defined by crystal symmetry. An approximate (isotropic) treatment of cell e.s.d.'s is used for estimating e.s.d.'s involving l.s. planes.
Refinement. Refinement of *F*^2^ against ALL reflections. The weighted *R*-factor *wR* and goodness of fit *S* are based on *F*^2^, conventional *R*-factors *R* are based on *F*, with *F* set to zero for negative *F*^2^. The threshold expression of *F*^2^ > σ(*F*^2^) is used only for calculating *R*-factors(gt) *etc*. and is not relevant to the choice of reflections for refinement. *R*-factors based on *F*^2^ are statistically about twice as large as those based on *F*, and *R*- factors based on ALL data will be even larger.

### Fractional atomic coordinates and isotropic or equivalent isotropic displacement parameters (Å^2^)

**Table:**

	*x*	*y*	*z*	*U*_iso_*/*U*_eq_	Occ. (<1)
Ni1	0.5000	0.0000	0.5000	0.03111 (13)	
S1	0.64988 (5)	0.40238 (4)	0.68338 (4)	0.04007 (17)	
O1	0.6512 (3)	0.42317 (15)	0.77146 (15)	0.0660 (8)	0.833 (4)
O2	0.5613 (3)	0.45099 (16)	0.6392 (2)	0.0928 (12)	0.833 (4)
O3	0.7645 (2)	0.41103 (15)	0.64593 (19)	0.0679 (9)	0.833 (4)
O1'	0.5600 (13)	0.4437 (7)	0.7240 (10)	0.071 (3)	0.167 (4)
O2'	0.6567 (14)	0.4346 (7)	0.5970 (8)	0.067 (3)	0.167 (4)
O3'	0.7703 (12)	0.4053 (8)	0.7150 (11)	0.070 (3)	0.167 (4)
O4	0.55301 (15)	0.12118 (10)	0.46049 (10)	0.0419 (4)	
O5	0.32791 (14)	0.04268 (11)	0.51454 (10)	0.0457 (4)	
H5B	0.2809	0.0148	0.5466	0.069*	
H5C	0.2878	0.0539	0.4707	0.069*	
O6A	0.8209 (4)	0.4456 (7)	0.8924 (4)	0.087 (2)	0.637 (18)
O6B	0.8230 (7)	0.4993 (10)	0.8633 (7)	0.084 (4)	0.363 (18)
H6B	0.7886	0.4576	0.8376	0.125*	
H6A	0.8896	0.4591	0.8752	0.125*	
N1	0.52928 (17)	0.02527 (12)	0.63186 (12)	0.0364 (4)	
H1A	0.4636	0.0100	0.6602	0.044*	
H1B	0.5881	−0.0098	0.6497	0.044*	
N2	0.54722 (19)	0.26684 (12)	0.43764 (13)	0.0439 (5)	
C1	0.61336 (19)	0.28983 (14)	0.67466 (13)	0.0342 (5)	
C2	0.49911 (19)	0.26121 (15)	0.68652 (16)	0.0413 (6)	
H2	0.4404	0.3010	0.7016	0.050*	
C3	0.4716 (2)	0.17309 (15)	0.67590 (15)	0.0405 (6)	
H3	0.3945	0.1539	0.6841	0.049*	
C4	0.5583 (2)	0.11367 (14)	0.65318 (14)	0.0340 (5)	
C5	0.6738 (2)	0.14216 (15)	0.64516 (16)	0.0435 (6)	
H5A	0.7332	0.1021	0.6326	0.052*	
C6	0.7012 (2)	0.22980 (15)	0.65571 (16)	0.0426 (6)	
H6	0.7790	0.2486	0.6501	0.051*	
C7	0.5020 (2)	0.19226 (16)	0.46400 (16)	0.0409 (6)	
H7	0.4264	0.1934	0.4868	0.049*	
C8	0.6644 (3)	0.26965 (19)	0.4022 (2)	0.0663 (8)	
H8A	0.7151	0.3036	0.4380	0.099*	
H8B	0.6613	0.2962	0.3475	0.099*	
H8C	0.6948	0.2110	0.3974	0.099*	
C9	0.4834 (3)	0.34922 (18)	0.4453 (2)	0.0702 (9)	
H9A	0.4083	0.3387	0.4715	0.105*	
H9B	0.4713	0.3740	0.3904	0.105*	
H9C	0.5284	0.3894	0.4790	0.105*	

### Atomic displacement parameters (Å^2^)

**Table:**

	*U*^11^	*U*^22^	*U*^33^	*U*^12^	*U*^13^	*U*^23^
Ni1	0.0330 (2)	0.0253 (2)	0.0350 (2)	−0.00230 (15)	−0.00010 (17)	0.00224 (16)
S1	0.0420 (3)	0.0321 (3)	0.0461 (4)	−0.0058 (2)	0.0025 (3)	−0.0025 (3)
O1	0.097 (2)	0.0512 (14)	0.0500 (15)	−0.0101 (13)	0.0151 (14)	−0.0190 (11)
O2	0.091 (2)	0.0413 (14)	0.146 (3)	−0.0075 (14)	−0.059 (2)	0.0242 (17)
O3	0.0698 (17)	0.0487 (13)	0.085 (2)	−0.0243 (12)	0.0369 (16)	−0.0132 (14)
O1'	0.078 (7)	0.035 (5)	0.099 (7)	−0.015 (5)	0.039 (6)	−0.029 (5)
O2'	0.085 (7)	0.039 (5)	0.075 (7)	−0.011 (5)	0.005 (6)	0.009 (5)
O3'	0.063 (6)	0.052 (5)	0.096 (7)	−0.018 (5)	−0.021 (6)	−0.007 (6)
O4	0.0504 (10)	0.0286 (8)	0.0467 (10)	−0.0046 (7)	0.0029 (8)	0.0029 (7)
O5	0.0366 (9)	0.0494 (10)	0.0512 (10)	0.0032 (8)	0.0009 (7)	0.0064 (8)
O6A	0.059 (2)	0.111 (5)	0.091 (4)	0.009 (3)	0.007 (2)	−0.036 (3)
O6B	0.074 (4)	0.088 (7)	0.089 (6)	−0.017 (4)	0.012 (4)	−0.048 (5)
N1	0.0410 (10)	0.0300 (9)	0.0380 (11)	−0.0026 (8)	−0.0014 (9)	0.0018 (8)
N2	0.0544 (12)	0.0298 (11)	0.0476 (12)	−0.0075 (9)	−0.0063 (10)	0.0053 (9)
C1	0.0374 (12)	0.0313 (11)	0.0338 (12)	−0.0037 (9)	0.0011 (10)	−0.0018 (9)
C2	0.0362 (12)	0.0353 (13)	0.0526 (15)	0.0016 (10)	0.0061 (11)	−0.0024 (11)
C3	0.0320 (11)	0.0388 (13)	0.0506 (15)	−0.0033 (10)	0.0042 (10)	−0.0004 (11)
C4	0.0393 (12)	0.0313 (12)	0.0315 (12)	−0.0020 (9)	−0.0023 (10)	0.0012 (9)
C5	0.0352 (12)	0.0363 (13)	0.0591 (16)	0.0026 (10)	0.0025 (11)	−0.0067 (11)
C6	0.0308 (11)	0.0404 (14)	0.0566 (15)	−0.0060 (10)	0.0027 (11)	−0.0052 (11)
C7	0.0448 (14)	0.0372 (14)	0.0407 (13)	−0.0053 (11)	−0.0031 (11)	0.0046 (11)
C8	0.072 (2)	0.0506 (17)	0.076 (2)	−0.0178 (14)	0.0161 (16)	0.0022 (15)
C9	0.072 (2)	0.0375 (15)	0.101 (3)	0.0028 (14)	−0.0144 (18)	0.0084 (16)

### Geometric parameters (Å, °)

**Table:**

Ni1—O4	2.0385 (15)	N1—H1A	0.9000
Ni1—O4^i^	2.0385 (15)	N1—H1B	0.9000
Ni1—O5^i^	2.0664 (15)	N2—C7	1.314 (3)
Ni1—O5	2.0664 (15)	N2—C8	1.442 (3)
Ni1—N1^i^	2.1579 (19)	N2—C9	1.452 (3)
Ni1—N1	2.1579 (19)	C1—C2	1.378 (3)
S1—O1'	1.359 (11)	C1—C6	1.383 (3)
S1—O2	1.431 (3)	C2—C3	1.387 (3)
S1—O3	1.434 (2)	C2—H2	0.9300
S1—O1	1.436 (2)	C3—C4	1.382 (3)
S1—O3'	1.454 (12)	C3—H3	0.9300
S1—O2'	1.461 (12)	C4—C5	1.383 (3)
S1—C1	1.767 (2)	C5—C6	1.379 (3)
O4—C7	1.228 (3)	C5—H5A	0.9300
O5—H5B	0.8499	C6—H6	0.9300
O5—H5C	0.8499	C7—H7	0.9300
O6A—O6B	0.940 (10)	C8—H8A	0.9600
O6A—H6B	0.9632	C8—H8B	0.9600
O6A—H6A	0.8491	C8—H8C	0.9600
O6B—H6B	0.8500	C9—H9A	0.9600
O6B—H6A	0.9898	C9—H9B	0.9600
N1—C4	1.426 (3)	C9—H9C	0.9600
			
O4—Ni1—O4^i^	180.0	O6A—O6B—H6A	52.1
O4—Ni1—O5^i^	88.41 (7)	H6B—O6B—H6A	88.7
O4^i^—Ni1—O5^i^	91.59 (7)	C4—N1—Ni1	115.77 (14)
O4—Ni1—O5	91.59 (7)	C4—N1—H1A	108.3
O4^i^—Ni1—O5	88.41 (7)	Ni1—N1—H1A	108.3
O5^i^—Ni1—O5	180.0	C4—N1—H1B	108.3
O4—Ni1—N1^i^	84.65 (7)	Ni1—N1—H1B	108.3
O4^i^—Ni1—N1^i^	95.35 (7)	H1A—N1—H1B	107.4
O5^i^—Ni1—N1^i^	88.85 (7)	C7—N2—C8	120.6 (2)
O5—Ni1—N1^i^	91.15 (7)	C7—N2—C9	121.7 (2)
O4—Ni1—N1	95.35 (7)	C8—N2—C9	117.7 (2)
O4^i^—Ni1—N1	84.65 (7)	C2—C1—C6	119.7 (2)
O5^i^—Ni1—N1	91.15 (7)	C2—C1—S1	121.02 (17)
O5—Ni1—N1	88.85 (7)	C6—C1—S1	119.27 (17)
N1^i^—Ni1—N1	180.0	C1—C2—C3	120.0 (2)
O1'—S1—O2	58.0 (8)	C1—C2—H2	120.0
O1'—S1—O3	146.3 (5)	C3—C2—H2	120.0
O2—S1—O3	112.5 (2)	C4—C3—C2	120.4 (2)
O1'—S1—O1	56.1 (8)	C4—C3—H3	119.8
O2—S1—O1	111.9 (2)	C2—C3—H3	119.8
O3—S1—O1	112.05 (17)	C3—C4—C5	119.3 (2)
O1'—S1—O3'	121.5 (9)	C3—C4—N1	121.1 (2)
O2—S1—O3'	144.2 (5)	C5—C4—N1	119.4 (2)
O3—S1—O3'	45.0 (6)	C6—C5—C4	120.3 (2)
O1—S1—O3'	69.2 (7)	C6—C5—H5A	119.9
O1'—S1—O2'	109.3 (9)	C4—C5—H5A	119.9
O2—S1—O2'	53.2 (6)	C5—C6—C1	120.2 (2)
O3—S1—O2'	62.0 (6)	C5—C6—H6	119.9
O1—S1—O2'	147.5 (5)	C1—C6—H6	119.9
O3'—S1—O2'	105.4 (9)	O4—C7—N2	124.3 (2)
O1'—S1—C1	108.1 (5)	O4—C7—H7	117.9
O2—S1—C1	107.36 (13)	N2—C7—H7	117.9
O3—S1—C1	105.55 (12)	N2—C8—H8A	109.5
O1—S1—C1	107.01 (12)	N2—C8—H8B	109.5
O3'—S1—C1	106.0 (5)	H8A—C8—H8B	109.5
O2'—S1—C1	105.3 (5)	N2—C8—H8C	109.5
C7—O4—Ni1	130.13 (16)	H8A—C8—H8C	109.5
Ni1—O5—H5B	120.0	H8B—C8—H8C	109.5
Ni1—O5—H5C	118.3	N2—C9—H9A	109.5
H5B—O5—H5C	105.0	N2—C9—H9B	109.5
O6B—O6A—H6B	53.0	H9A—C9—H9B	109.5
O6B—O6A—H6A	67.0	N2—C9—H9C	109.5
H6B—O6A—H6A	90.6	H9A—C9—H9C	109.5
O6A—O6B—H6B	64.9	H9B—C9—H9C	109.5

Symmetry codes: (i) −*x*+1, −*y*, −*z*+1.

### Hydrogen-bond geometry (Å, °)

**Table:**

*D*—H···*A*	*D*—H	H···*A*	*D*···*A*	*D*—H···*A*
N1—H1A···O1^ii^	0.90	2.15	2.992 (3)	156
N1—H1B···O3^iii^	0.90	2.06	2.919 (3)	160
O5—H5B···O6A^ii^	0.85	1.84	2.685 (5)	174
O5—H5B···O6B^ii^	0.85	1.87	2.669 (8)	156
O5—H5C···O3^iv^	0.85	1.95	2.743 (3)	155
O6A—H6A···O2^v^	0.85	1.96	2.768 (6)	158
O6B—H6B···O1	0.85	1.95	2.694 (8)	146

Symmetry codes: (ii) −*x*+1, *y*−1/2, −*z*+3/2; (iii) −*x*+3/2, *y*−1/2, *z*; (iv) *x*−1/2, −*y*+1/2, −*z*+1; (v) *x*+1/2, *y*, −*z*+3/2.
